# The Impact of Bisphenol A and Triclosan on Immune Parameters in the U.S. Population, NHANES 2003–2006

**DOI:** 10.1289/ehp.1002883

**Published:** 2010-11-29

**Authors:** Erin M. Rees Clayton, Megan Todd, Jennifer Beam Dowd, Allison E. Aiello

**Affiliations:** 1 Department of Epidemiology and Center for Social Epidemiology and Population Health, University of Michigan School of Public Health, Ann Arbor, Michigan, USA; 2 Institute for Demographic Research, City University of New York, New York, New York, USA; 3 Woodrow Wilson School of Public and International Affairs and Office of Population Research, Princeton University, Princeton, New Jersey, USA; 4 School of Public Health, Hunter College, City University of New York, New York, New York, USA

**Keywords:** allergies, bisphenol A, cytomegalovirus, endocrine-disrupting compounds, NHANES, triclosan

## Abstract

**Background:**

Exposure to environmental toxicants is associated with numerous disease outcomes, many of which involve underlying immune and inflammatory dysfunction.

**Objectives:**

To address the gap between environmental exposures and immune dysfunction, we investigated the association of two endocrine-disrupting compounds (EDCs) with markers of immune function.

**Methods:**

Using data from the 2003–2006 National Health and Nutrition Examination Survey, we compared urinary bisphenol A (BPA) and triclosan levels with serum cytomegalovirus (CMV) antibody levels and diagnosis of allergies or hay fever in U.S. adults and children ≥ 6 years of age. We used multivariate ordinary least squares linear regression models to examine the association of BPA and triclosan with CMV antibody titers, and multivariate logistic regression models to investigate the association of these chemicals with allergy or hay fever diagnosis. Statistical models were stratified by age (< 18 years and ≥ 18 years).

**Results:**

In analyses adjusted for age, sex, race, body mass index, creatinine levels, family income, and educational attainment, in the ≥ 18-year age group, higher urinary BPA levels were associated with higher CMV antibody titers (*p* < 0.001). In the < 18-year age group, lower levels of BPA were associated with higher CMV antibody titers (*p* < 0.05). However, triclosan, but not BPA, showed a positive association with allergy or hay fever diagnosis. In the < 18-year age group, higher levels of triclosan were associated with greater odds of having been diagnosed with allergies or hay fever (*p* < 0.01).

**Conclusions:**

EDCs such as BPA and triclosan may negatively affect human immune function as measured by CMV antibody levels and allergy or hay fever diagnosis, respectively, with differential consequences based on age. Additional studies should be done to investigate these findings.

Exposure to environmental toxicants is associated with numerous disease outcomes, many of which involve underlying immune and inflammatory dysfunction (Dietert et al. 2010). Mounting epidemiological evidence suggests that proper function of the immune system is a major determinant of health across the life course (Dietert et al. 2010). For example, those with childhood asthma may have an elevated risk of developing lung cancer (Dietert et al. 2010), and among individuals ≥ 87 years old, immune dysregulation has been linked with dementia (Katsel et al. 2009). Exposure to environmental toxicants, therefore, may cause life-long changes in response to infectious agents, in immune homeostasis, and in overall physical and mental health (Dietert et al. 2010).

One group of environmental toxicants affecting immune system function is endocrine-disrupting compounds (EDCs) (Chalubinski and Kowalski 2006). In 1996, the U.S. Congress first recognized EDCs as a public health concern when it mandated that the U.S. Environmental Protection Agency (EPA) develop a way to identify and monitor EDCs in the environment (Endocrine Society 2009). EDCs are substances found in food, personal care and other consumer products, pharmaceuticals, and the environment that have deleterious effects on human health, homeostasis, metabolism, and reproduction (Diamanti-Kandarakis et al. 2009). Synthetic EDCs form a diverse group of chemicals primarily used in pesticides, industrial chemicals, pharmaceuticals, and personal care products (Chalubinski and Kowalski 2006). Sources of exposure to EDCs can vary widely, ranging from occupational to *in utero* exposures (Barr et al. 2007). The stability (among other characteristics) of many EDCs that makes these substances beneficial for industry also means that high levels of these chemicals can be detected in water, air, and soil even years after being banned from use, which results in extended windows of time during which humans may be exposed (Diamanti-Kandarakis et al. 2009). Although the exact physiological mechanisms of action are unknown for many EDCs, they mimic or affect hormones (Diamanti-Kandarakis et al. 2009). Despite increasing research on the effects of EDCs on human health, controversy surrounds the issue of what concentrations—if any—of these chemicals are safe for human use.

The industrial chemical bisphenol A (BPA) is a prevalent EDC in human products and environments (Vandenberg et al. 2010; Welshons et al. 2006). BPA (4,4′-isopropylidenediphenol) is used primarily as a component of polycarbonate plastics, epoxy resins, and dental sealants, with > 800 million kg BPA produced each year in the United States alone (Diamanti-Kandarakis et al. 2009). Among the many health effects associated with BPA exposure, this chemical has been linked with abnormal male and female reproductive organ development in animals (Vandenberg et al. 2010) and sperm anomalies in humans (Meeker et al. 2010).

According to U.S. EPA guidelines, safe levels of BPA intake are 50 μg/kg body weight/day, assuming that the main source of exposure is from ingestion. In a recent analysis of 2005–2006 National Health and Nutrition Examination Survey (NHANES) data, the median daily intake of BPA in the United States was estimated to be 0.034 μg/kg/day, which is well below the U.S. EPA guidelines of 50 μg/kg/day (Lakind and Naiman 2010). Nevertheless, studies suggest that the U.S. EPA guidelines and the conclusion that current dietary intake levels are safe should be reevaluated (Vandenberg et al. 2010). First, low-dose exposures to BPA (> 200 times below the U.S. EPA recommended dose) in pregnant mice negatively affected mammary gland development of female offspring by increasing ductal area and extension, promoting fat pad maturation, and decreasing epithelial cell size (Vandenberg et al. 2007). In addition, tumor-promoting agonistic effects of BPA on a mutant androgen receptor in a human prostate adenocarcinoma cell line were more pronounced at low doses than at higher doses (Keri et al. 2007). Therefore, EDCs may possess nonlinear dose–response dynamics, with low doses causing greater abnormalities. Second, urinary BPA levels have not been shown to decline consistently with increasing fasting times; this finding suggests that dietary exposure may not be the only important source of exposure, or that BPA is not cleared rapidly from the body (Stahlhut et al. 2009; Vandenberg et al. 2010). Thus, a need exists to better understand how chronic exposure to low doses of BPA may affect human health.

Another prevalent EDC, triclosan (2,4,4′-trichloro-2′-hydroxydiphenyl ether), was first introduced in the 1960s for use in personal care products as an antimicrobial and preservative. Since then, triclosan has been added to countless consumer products, including, but not limited to, hand soaps, laundry detergents, toothpastes, wound disinfection solutions, deodorants, facial tissues, plastic kitchen utensils, medical devices, and toys [Centers for Disease Control and Prevention (CDC) 2010; U.S. EPA 2010]. The proliferation of triclosan as an “antibacterial” ingredient coincides with mounting evidence of its bioaccumulation and persistence in human fat tissue, umbilical cord blood, human breast milk, and urine (Smith and Lourie 2009). The most likely routes of exposure to triclosan are ingestion and absorption through the skin (Calafat et al. 2008). The CDC, in a study conducted from 2003 through 2004 among a representative sample of the U.S. general population ≥ 6 years of age, found triclosan at unadjusted concentrations of 2.4–3,790 μg/L in the urine of 75% of Americans (Calafat et al. 2008). The significance of this finding remains unknown.

Despite the initial safety data demonstrating triclosan’s tolerance by humans, more recent studies have found that it may have detrimental effects on the central nervous system and may be linked to allergies and asthma (Glaser 2004; Kepner 2004–2005). Triclosan was first labeled as an EDC in 2006 when Veldhoen et al. (2006) demonstrated that triclosan (at concentrations as low as 0.15 μg/L) decreased body weight and accelerated thyroid hormone–mediated hind limb development in the bullfrog *Rana catesbeiana*. Thus, triclosan may not mimic thyroid hormones but may influence how they act, affecting the development and metabolism of not only frogs but all species that depend on thyroid hormone signaling, including humans.

The ability of chemicals such as BPA and triclosan to interfere with the endocrine system, and the interactions between the endocrine and immune systems, suggests that EDCs may also target immune function (Ahmed 2000; Chalubinski and Kowalski 2006). Immunological changes, including increased CD4^+^:CD8^+^ratios, decreased proliferation of peripheral blood mononuclear cells, and increased frequency of autoantibodies, have all been observed in humans with occupational exposures to pesticides containing EDCs (Ahmed 2000; McConnachie and Zahalsky 1992; Rosenberg et al. 1999; Straube et al. 1999). Yet, no epidemiological data have been examined to determine whether BPA or triclosan influences immune function. Natural estrogen (17β-estradiol) has been shown to disrupt morphology of the thymus and bone marrow, protect the main cells of the immune system (T, B, and antigen-presenting cells) from apoptosis, and regulate the synthesis of antibodies in the serum and female genital tract (Ahmed 2000; Donner et al. 1999; Grossman 1984; Verthelyi and Ahmed 1998; Wira and Sandoe 1987). It is therefore plausible that EDCs such as BPA and triclosan, which possess estrogenic activity (Ishibashi et al. 2004), may exert similar effects within the body.

Laboratory studies have also shown that EDCs, such as BPA, diethylhexyl phthalate, and 4-*tert*-octylphenol, can be involved in the development of allergies by increasing interleukin-4 production and IgE levels (Chalubinski and Kowalski 2006; Lee et al. 2003). For triclosan, some have hypothesized that using this antimicrobial ingredient may increase allergies among humans via the “hygiene hypothesis.” The hygiene hypothesis, first proposed by Strachan (1989), has evolved into a potential explanation for the increase in allergic and autoimmune diseases observed in recent decades, particularly in developed countries. This hypothesis attributes the rise in allergies to the increase in clean, hygienic living environments and subsequent decrease in exposure to infectious agents (Okada et al. 2010). Exposure to triclosan and other antibacterial agents may lead to an excessively clean environment that disrupts the usual development of the immune system by eliminating or changing the commensal microbiota that normally help to shape the immune system (Levy 2001; Okada et al. 2010).

To begin to address the gaps that exist in understanding how environmental exposures and immune dysfunction are related, we investigated the association of two EDCs with markers of immune function. We hypothesized that exposure to BPA and triclosan may lead to impaired immune function and increased allergies. Using data from the 2003–2006 NHANES, we examined whether urinary levels of BPA and triclosan were associated with reported allergies or measured serum antibody titers to a common pathogen of the herpes virus family, cytomegalovirus (CMV). Increased CMV antibody titers are considered a marker of altered cell-mediated immune function (Dowd and Aiello 2009; Koch et al. 2006; McDade et al. 2000; Stowe et al. 2007), and high antibody levels indicate that the cellular arm of the immune response is less effective in keeping the virus in a persistent latent state (Roberts et al. 2010). CMV antibody levels are useful as a marker of immune function because primary infection with this virus often occurs at a very young age, the virus persists in the infected host for life, and containment of the virus becomes a top priority for the immune system (Dowd and Aiello 2009; Koch et al. 2006). This study provides an important first step toward understanding how two prevalent EDCs, BPA and triclosan, may interact with human immune function.

## Materials and Methods

### Data source

We used survey and laboratory data from the 2003–2006 U.S. NHANES for this study (CDC 2011a, 2011b). NHANES is a U.S. nationally representative cross-sectional survey, with interview, examination, and laboratory data collected continuously and released in 2-year waves. Participants were chosen via a stratified multistage sampling method, with oversamples of African Americans, Mexican Americans, and persons > 60 years of age.

### Outcomes

CMV-specific IgG was measured in the 2003–2004 wave for respondents 6–49 years of age using an enzyme-linked immunosorbent assay (ELISA) by Quest International Inc. (Miami, FL). Serum samples near the ELISA cutoff were confirmed with a second ELISA assay by bioMérieux Inc. (Durham, NC). When these two tests disagreed, an immunofluorescent assay (IFA) from Bion International, Inc. (Des Plaines, IL) was performed, and the result of the IFA was given as the final result. All assays were carried out according to manufacturer guidelines. CMV-specific IgG optical density was reported [measured in arbitrary units (AU) per milliliter] and used as a continuous outcome variable. CMV seropositivity was defined by NHANES based on this optical density measure (CDC 2003a).

A diagnosis of allergy or hay fever was determined by two questions in the NHANES interview. Respondents were asked, “Has a doctor or other health professional ever told you that you have allergies/hay fever?” A respondent was coded as having allergies or hay fever if the response to either question was yes.

### Predictors

Urinary BPA and triclosan were measured in a random subsample of one-third of participants > 6 years of age (*n* = 5,250). Aliquots (100 μL) of single-void urine specimens (spot urine samples) were analyzed using solid-phase extraction coupled online to high-performance liquid chromatography and tandem mass spectrometry. Analysis was conducted by the Division of Environmental Health Laboratory Sciences, National Center for Environmental Health, CDC, using methods previously described (Ye et al. 2005). The lower limit of detection for BPA was 0.36 ng/mL in 2003–2004 and 0.4 ng/mL in 2005–2006; the lower limit of detection for triclosan was 2.27 ng/mL in 2003–2004 and 2.3 ng/mL in 2005–2006 (CDC 2003b).

### Covariates

CDC calculated body mass index (BMI) as kilograms per meters squared-from measured height and weight during the exam and measured urinary creatinine in micrograms per deciliter and included to control for the level of dilution of urine analytes. We coded family income as the midpoint of each of 11 reported income categories. Income was adjusted to year 2000 dollars using the Consumer Price Index (Bureau of Labor Statistics 2011). For the top-coded income category (> $65,000), we used the median family income for those with income > $65,000 (according to the 2000 current population survey) (Bureau of Labor Statistics and Bureau of the Census 2011). Income was also divided into quartiles within each wave. We coded race/ethnicity as non-Hispanic white, non-Hispanic black, Mexican American, and other race/ethnic groups (including other Hispanic groups). Educational attainment of the household reference person was coded in three categories: less than high school, completed high school, and more than high school.

### Sample

**Figure 1:**
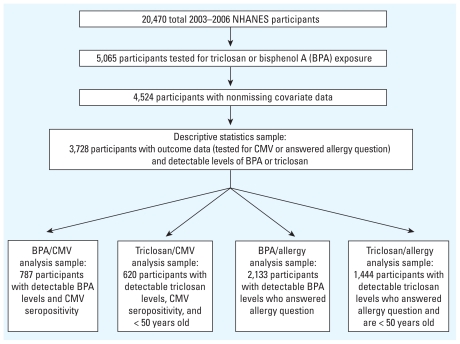
Analytic sample creation.

Triclosan and BPA measures were available for 5,065 respondents; 541 respondents (10.6%) were missing data on one or more covariates, with an additional 796 respondents missing data on continuous CMV or allergy diagnosis, for a final sample of 3,728 respondents. Regressions of CMV were limited to CMV-seropositive respondents with nonmissing CMV optical density measures (available in the 2003–2004 wave). Regressions were also limited to respondents with detected levels of BPA (when BPA was a predictor) or triclosan (when triclosan was a predictor). Regressions including triclosan as a predictor were further limited to respondents no more than 50 years of age at the time of the exam because triclosan was not introduced into the community setting until the 1960s, with wide-scale use in consumer products occurring in the late 1980s and 1990s (Coulborn et al. 2010). This age restriction allowed us to analyze a population that would have had ample opportunity to be exposed to triclosan throughout the entire life span. See Figure 1 for details on analytic sample breakdown.

### Statistical analysis

Ordinary least squares (OLS) regressions were used to model continuous CMV; logistic regressions were used to model allergies/hay fever. Two models were used to examine the association of triclosan/BPA with each outcome of interest: Model 1 included age, sex, creatinine, and race as covariates; model 2 included all of these plus BMI, family income, and educational attainment. Models were stratified by age (< 18 vs. ≥ 18 years). We chose to examine the effects of triclosan and BPA on immune function separately for children and adults, because of potential differences in length of exposure to triclosan or BPA, immune system development, and differences in the prevalence of CMV infection, antibody levels, and allergies among children versus adults (Arbes et al. 2005; Bate et al. 2010; Calafat et al. 2008; Dowd and Aiello 2009; LaKind and Naiman 2010; Vandenberg et al. 2010). All models used sampling weights and adjusted for the NHANES complex survey design. Analyses were conducted using STATA (version 10.0; StataCorp LP, College Station, TX).

## Results

### Demographic characteristics of the study population

Table 1 summarizes the physical and sociodemographic characteristics of the study population (*n* = 3,728). The mean concentration of urinary triclosan was > 25 times higher than that of BPA (116.3 vs. 4.4 ng/mL). Of the 46.7% of the population testing positive for CMV IgG antibody, the mean antibody level was 2.1 AU/mL. Approximately one-third of the population (33.8%) reported that a doctor had diagnosed them with allergies or hay fever at some point during their life.

### Triclosan and BPA concentrations by sociodemographic characteristics

We next examined whether mean triclosan and BPA concentrations differed by sociodemographic group (Table 2). We observed no statistically significant differences in mean triclosan or BPA levels between CMV-seropositive and CMV-seronegative individuals, between those with and without allergies or hay fever, or across levels of educational attainment of the head of household. Mexican Americans had the highest mean concentration of triclosan (160.59 ng/mL), whereas non-Hispanic blacks had the highest mean concentration of BPA (5.38 ng/mL). Although we found no differences in mean BPA concentrations between males and females, males had significantly higher mean triclosan levels than did females (136.76 vs. 95.38 ng/mL). Interestingly, both triclosan and BPA concentrations differed across family income quartiles, but the trend was in the opposite direction: Those in the lowest family income quartile had significantly higher mean BPA concentrations than did those in the highest quartile (5.73 vs. 3.75 ng/mL) but lower mean concentrations of triclosan (94.80 vs. 138.06 ng/mL).

### Association between triclosan and BPA, and CMV antibody levels

Triclosan levels were not significantly associated with CMV antibody levels in either age group, regardless of whether the models were minimally adjusted (for age, sex, race, and creatinine levels) or fully adjusted (for additional characteristics such as BMI, family income, and educational attainment; Table 3). Among those ≥ 18 years of age, increasing age, and female were associated with higher CMV antibody levels; adjusting for income and education had almost no effect on these associations. For BPA, increasing concentrations of BPA were associated with increasing CMV antibody levels in those ≥ 18 years of age in both minimally and fully adjusted models (Table 4). In children and adolescents < 18 years of age, we found an inverse relation between BPA levels and CMV antibody titers when controlling for BMI and socioeconomic position (SEP).

### Association between triclosan and BPA, and allergies

Higher triclosan levels were associated with greater odds of having been diagnosed with allergies or hay fever (Table 5). We observed this association in both age groups, but it was statistically significant only for those < 18 years old [odds ratio (OR) = 1.257; *p* < 0.01]. We found no association of BPA with allergy or hay fever diagnosis (Table 6). Males had higher odds of allergies in the younger age group than did females, with the reverse pattern in adults.

## Discussion

Data on the immune consequences of exposure to EDCs are lacking. With the interaction of the endocrine, immune, and nervous systems, it is likely that EDCs may induce pathophysiology not only in the endocrine system but also in other human body systems. Given the crucial role the immune system plays in determining overall health status (Dietert et al. 2010), it is essential to begin to address the impact of prevalent EDCs, including BPA and triclosan, on immune parameters.

Our analyses adjusting for physical, demographic, and socioeconomic characteristics indicated that higher levels of urinary BPA, but not triclosan, were associated with higher CMV antibody levels, suggesting that the immune systems of individuals with higher BPA exposure are less able to control chronic infections. One potential mechanistic explanation for this association is that BPA may inhibit macrophage function (Segura et al. 1999). An important first step in the phagocytic function of macrophages is adherence, and *in vitro* exposure of rat peritoneal macrophages to BPA has demonstrated suppressed macrophage adherence (Segura et al. 1999). CMV persists in immune cells of the myeloid lineage (Taylor-Wiedeman et al. 1991), such as mature monocytes, which can go on to develop into macrophages. If BPA disrupts the function of macrophages, it may be easier for CMV to survive and replicate, increasing the need for the host to increase antibody production to counteract CMV reactivation.

In a recently published analysis of 2003–2004 NHANES data, LaKind and Naiman (2010) reported significantly higher urinary concentrations of BPA in the youngest age groups of their study population, compared with older age groups. The study also found higher urinary BPA levels in men compared with women. We observed a similar trend by sex in our analyses, but this difference did not reach statistical significance. Consistent with results from LaKind and Naiman (2010), we found that non-Hispanic blacks had significantly higher urinary BPA levels than did non-Hispanic whites.

It is unclear why BPA was positively associated with CMV antibody levels in adults but negatively associated with CMV at younger ages. The physiological consequences of BPA exposure may vary based on the quantity, duration, and/or timing of exposure. Short exposures to BPA—and potentially other EDCs—may provide a measure of positive immune stimulation, whereas longer exposures could tip the balance toward immune dysfunction. One example of an EDC that has been shown to possess both beneficial and harmful effects is the isoflavone genistein. Although naturally occurring (unlike BPA), genistein has been identified as an EDC because of its ability to exhibit estrogenic activity, its association with reproductive problems in animals, and its link to adenocarcinomas and mammary tumors in rodents. A potential benefit of isoflavones such as genistein is antiviral properties against a variety of virus families in both *in vitro* and *in vivo* studies (Andres et al. 2009). The mechanism of this antiviral activity is unknown, but isoflavones have been shown to exert effects on both the virus and the host (Andres et al. 2009). Thus, BPA may initially pose a larger threat to CMV than to the human host, with the relation shifting over time as immunosenescent changes occur with aging, leading to immune dysfunction and higher CMV antibody levels. Further studies are needed to address the potential immune-boosting effects BPA may have in naive immune systems, as well as the detrimental effects shown here on adults with developed immune systems.

In individuals < 18 years of age, higher concentrations of urinary triclosan were associated with greater odds of having been diagnosed with allergies even after adjusting for age, sex, BMI, race, and family income and education. The “hygiene hypothesis” argues that the developing human immune system needs exposure to commensal microorganisms for proper stimulation of T helper 1 (T_H_1) and T_H_2 cells (Okada et al. 2010). Most individuals with allergies have an imbalance in T_H_1 and T_H_2 cells, with more T_H_2 cells that promote antibody production (Levy 2001). It is possible that exposure to triclosan, particularly at times during the life course when the immune system is developing, may modify immunological responses to microorganisms in such a way that imbalances between T_H_1 and T_H_2 cells are created. These T-cell imbalances may stimulate autoimmune reactions and allergies.

Calafat et al. (2008) reported that almost 75% of the 2003–2004 NHANES participants studied had measurable amounts of triclosan in their urine. Triclosan concentrations ranged from 2.4 to 3,790 μg/L, with the highest levels occurring in young adults in their 20s and those of higher SEP (Calafat et al. 2008). In the analyses we present here, higher SEP (as measured by family income levels) was also associated with higher urinary triclosan concentrations. In multivariate logistic regression models, individuals living in households where the head of household was more educated had greater odds of ever having been diagnosed with allergies and/or hay fever. Although asthma and wheezing are more common in persons of lower SEP (McHugh et al. 2009), hay fever and eczema seem to be more common in non-Hispanic whites and persons of higher SEP (Litonjua et al. 2005). Thus, it may be that triclosan exposure is associated more with atopic disorders often seen in high-SEP populations than it is with asthma-related allergies that are more common among those of lower SEP.

Limitations to the present study include its cross-sectional nature, which prohibits any investigation of temporality and therefore does not provide a definitive causal pathway for triclosan or BPA as they relate to alterations in immune function. Further biological studies examining the pathways by which these chemicals may influence the human immune system are warranted. We also did not address the impact of mixture effects, so it is possible that triclosan or BPA alone may not be at the origin of the described effects. In addition, we determined body levels of these chemicals by urinary concentrations, which may not be as physiologically relevant as the free concentrations of triclosan or BPA in the bloodstream (Vandenberg et al. 2010). Furthermore, we did not consider important factors that may affect the relation of triclosan and BPA to immune function, such as psychosocial stress and genetics, because of limitations in the data collected. Given that the allergy/hay fever outcome was self-reported from previous physician diagnosis, some bias may exist in this variable if participants who have allergies or hay fever have not seen a doctor and been diagnosed. Although CMV antibody levels have proven to be an important indicator of cellular immune function and allergy or hay fever provides a good indication of the T_H_2 arm of the immune system, few other measures of immune function were available in NHANES for the age range included in our analyses. Future studies should examine cellular phenotypic markers and function to assess whether the results here are supported by a broader set of markers involved in immune alterations. Finally, the sample sizes for each age-stratified model limit our power to detect very small effects on the immune parameters of interest. Because of these limitations, more studies are needed to address the impact of BPA and triclosan on immune function and duplicate the findings we report.

## Conclusions

This novel study addresses the need to improve our understanding of how EDCs affect human health, particularly immune function. Using data from a nationally representative sample, this study is the first to our knowledge that demonstrates an association between BPA levels and CMV antibody levels, and one of the first to examine associations between triclosan concentrations and allergy or hay fever diagnosis. These results suggest that EDCs such as BPA and triclosan negatively alter immune function over the life course, in addition to posing a threat to endocrine and reproductive function. There is an urgent need to identify the underlying mechanisms of EDC-mediated immune dysfunction. Future studies should seek to elucidate these mechanisms, as well as how the timing and quantity of exposure to EDCs such as triclosan and BPA over the life course influence immune function and later-life disease susceptibility and severity. In addition, research in the fields of environmental health and infectious disease should consider how combined exposures to environmental toxicants and infectious agents may alter immune function and subsequent disease risk.

## Figures and Tables

**Table 1:** Estimated population means and frequencies for demographic characteristics of study population, NHANES 2003–2006.

Characteristic	Weighted mean or percentage (95% CI)	Linearized SE	*n*
Age (years)	35 (33.36–35.82)	0.60	3,728
Male (%)	50.5 (48–53)	1.10	3,728
BMI (kg/m^2^)	26.6 (26.13–27.05)	0.22	3,728
Lab measure			
Triclosan (ng/mL)	116.3 (105.43–127.11)	5.31	3,728
BPA (ng/mL)	4.4 (4.07–4.82)	0.18	3,728
CMV seropositive (%)	46.7 (42–52)	2.40	1,496
CMV-antibody level (if seropositive)	2.1 (1.93–2.17)	0.06	805
Creatinine (mg/dL)	134.9 (130.27–139.61)	2.29	3,728
Ever diagnosed with allergies or hay fever (%)	33.8% (31–36)	1.10	2,232
Race (%)			
Non-Hispanic white	68.3 (63.3–72.9)	2.40	1,457
Non-Hispanic black	12.9 (1–16.5)	1.60	1,073
Mexican American	9.7 (7.5–12.5)	1.20	933
Other race/ethnic groups	9.1 (7.4–0.11.1)	0.90	265
Family income [US$(2000)]	51,706 (48,809–54,603)	1,418	3,728
Family income quartile [US$(2000)]			
1	8,985		894
2	25,108		1,153
3	47,005		736
4	96,102		945
Head of household educational attainment (%)			
< High school	17.2 (15.3–19.2)	1.00	1,044
Completed high school	24.8 (22.6–0.27.2)	1.10	898
> High school	58.0 (54.8–61.2)	1.60	1,786

**Table 2:** Estimated mean triclosan and BPA levels, by sociodemographic characteristics, NHANES 2003–2006.

	Triclosan		BPA	
Characteristic	Mean (ng/mL)	*p*-Value	Mean (ng/mL)	*p*-Value
CMV		0.741		0.235

Seropositive	103.63		5.53	
Seronegative	99.38		4.83	

Ever diagnosed with allergies or hay fever		0.814		0.633

Yes	122.76		3.78	
No	127.53		4.09	

Sex		0.008		0.451

Male	136.76		4.58	
Female	95.38		4.31	

Race		0.095		< 0.001

Non-Hispanic white	110.56		4.04	
Non-Hispanic black	110.74		5.38	
Mexican American	160.59		5.32	
Other race/ethnic groups	119.56		5.21	

Family income quartile		0.119		0.001

1	94.80		5.73	
2	103.94		4.61	
3	113.41		4.37	
4	138.06		3.75	

Head of household educational attainment		0.608		0.629

< High school	121.01		4.51	
Completed high school	103.55		4.69	
> High school	120.31		4.32	

Mean triclosan and BPA levels are weighted. *p*-Values are from a Wald test of the difference between these weighted means.

**Table 3:** Association between triclosan levels and immune function measured by CMV antibody levels, NHANES 2003–2004.

	Model 1		Model 2	
Independent variable	< 18 years of age (*n* = 252)	≥ 18 years of age (*n* = 368)	< 18 years of age (*n* = 252)	≥ 18 years of age (*n* = 368)
Ln(triclosan) (ng/mL)	0.003 (0.046)	−0.024 (0.031)	0.012 (0.046)	−0.022 (0.032)
Age (years)	−0.026 (0.014)	0.020 (0.004)***	−0.025 (0.015)	0.021 (0.004)***
Male	−0.093 (0.136)	−0.316 (0.074)***	−0.130 (0.127)	−0.311 (0.079)**
BMI (kg/m^2^)			0.005 (0.009)	−0.003 (0.008)
Creatinine (mg/dL)	0.001 (0.001)	0.001 (0.001)	0.001 (0.001)	0.000 (0.001)
Race				
Non-Hispanic black	0.039 (0.101)	0.123 (0.097)	0.077 (0.147)	0.046 (0.111)
Mexican American	0.028 (0.146)	0.093 (0.114)	0.109 (0.209)	−0.045 (0.115)
Other race/ethnic groups	−0.092 (0.252)	−0.081 (0.168)	−0.003 (0.260)	−0.136 (0.183)
Family income quartile				
2			−0.176 (0.150)	−0.109 (0.133)
3			−0.276 (0.215)	−0.122 (0.117)
4			−0.063 (0.207)	−0.353 (0.176)
Head of household education				
Completed high school			0.193 (0.185)	−0.095 (0.147)
> High school			0.121 (0.163)	−0.096 (0.161)

Data are β-coefficients (SEs) for each independent variable in the regression models. Models are OLS regressions with CMV antibody level as the dependent variable, estimated among respondents with detected levels of triclosan. Only respondents < 50 years of age are included. Model 1 includes age, sex, creatinine, and race as covariates; model 2 includes all of these plus BMI, family income, and educational attainment.

** *p* < 0.01.

*** *p* < 0.001.

**Table 4:** Association between BPA levels and immune function measured by CMV antibody levels, NHANES 2003–2004.

	Model 1		Model 2	
Independent variable	< 18 years of age (*n* = 335)	≥ 18 years of age (*n* = 452)	< 18 years of age (*n* = 335)	≥ 18 years of age (*n* = 452)
Ln(BPA) (ng/mL)	−0.104 (0.053)	0.186 (0.034)***	−0.113 (0.047)*	0.158 (0.035)***
Age (years)	−0.011 (0.013)	0.017 (0.004)***	−0.012 (0.012)	0.018 (0.004)***
Male	−0.151 (0.117)	−0.250 (0.078)**	−0.154 (0.109)	−0.239 (0.078)**
BMI (kg/m^2^)			0.004 (0.008)	0.001 (0.006)
Creatinine (mg/dL)	0.001 (0.001)*	−0.001 (0.001)	0.001 (0.001)*	−0.001 (0.001)
Race				
Non-Hispanic black	0.179 (0.086)	0.158 (0.082)	0.145 (0.103)	0.095 (0.093)
Mexican American	0.105 (0.084)	0.128 (0.108)	0.090 (0.097)	−0.006 (0.115)
Other race/ethnic groups	0.288 (0.150)	−0.111 (0.141)	0.294 (0.154)	−0.211 (0.168)
Family income quartile				
2			−0.275 (0.134)	−0.055 (0.101)
3			−0.275 (0.164)	−0.166 (0.099)
4			−0.107 (0.138)	−0.305 (0.132)*
Head of household education				
Completed high school			0.070 (0.148)	−0.136 (0.108)
> High school			−0.015 (0.105)	−0.136 (0.154)

Data are β-coefficients (SEs) for each independent variable in the regression models. Models are OLS regressions with CMV antibody level as the dependent variable. The model is estimated among respondents with detected levels of BPA. Model 1 includes age, sex, creatinine, and race as covariates; model 2 includes all of these plus BMI, family income, and educational attainment.

* *p* < 0.05.

** *p* < 0.01.

*** *p* < 0.001.

**Table 5:** Association between triclosan levels and immune function measured by allergies or hay fever diagnosis: NHANES 2003–2006.

	Model 1		Model 2	
Independent variable	< 18 years of age (*n* = 678)	≥ 18 years of age (*n* = 766)	< 18 years of age (*n* = 678)	≥ 18 years of age (*n* = 766
Ln(triclosan) (ng/mL)	1.245 (0.109)*	1.004 (0.075)	1.257 (0.097)**	0.996 (0.074)
Age (years)	1.043 (0.029)	1.015 (0.008)	1.023 (0.029)	1.014 (0.009)
Male	1.396 (0.239)	0.624 (0.148)	1.379 (0.229)	0.631 (0.151)
BMI (kg/m^2^)			1.024 (0.019)	1.004 (0.014)
Creatinine (mg/dL)	0.999 (0.001)	0.999 (0.001)	0.999 (0.002)	0.999 (0.002)
Race				
Non-Hispanic black	1.199 (0.313)	0.781 (0.201)	1.303 (0.354)	0.803 (0.223)
Mexican American	0.439 (0.135)*	0.578 (0.130)*	0.588 (0.190)	0.755 (0.202)
Other race/ethnic groups	1.202 (0.417)	0.807 (0.261)	1.226 (0.456)	0.880 (0.269)
Family income quartile				
2			0.691 (0.334)	1.216 (0.349)
3			0.498 (0.146)*	0.801 (0.298)
4			0.760 (0.295)	1.099 (0.351)
Head of household education				
Completed high school			2.488 (0.855)*	1.638 (0.568)
> High school			3.107 (1.209)*	2.198 (0.665)*

Data are ORs (SEs) for allergy or hay fever diagnosis among unit changes (or categories) of the independent variable. Models are logistic regressions with dependent variable ever diagnosed with allergies or hay fever, estimated among respondents with detected levels of triclosan. Only respondents < 50 years of age are included. Model 1 includes age, sex, creatinine, and race as covariates; model 2 includes all of these plus BMI, family income, and educational attainment.

* *p* < 0.05.

** *p* < 0.01

**Table 6:** Association between BPA levels and immune function measured by allergies or hay fever diagnosis: NHANES 2003–2006.

	Model 1		Model 2	
Independent variable	< 18 years of age (*n* = 793)	≥ 18 years of age (*n* = 1,340)	< 18 years of age (*n* = 793)	≥ 18 years of age (*n* = 1,340)
Ln(BPA) (ng/mL)	0.910 (0.113)	0.896 (0.098)	0.933 (0.123)	0.903 (0.094)
Age (years)	1.045 (0.032)	1.001 (0.005)	1.029 (0.035)	1.003 (0.005)
Male	1.679 (0.262)**	0.707 (0.124)	1.632 (0.236)**	0.692 (0.123)
BMI (kg/m^2^)			1.023 (0.016)	1.006 (0.010)
Creatinine (mg/dL)	1.000 (0.002)	1.000 (0.001)	1.000 (0.002)	1.000 (0.001)
Race				
Non-Hispanic black	1.085 (0.256)	0.718 (0.121)	1.167 (0.292)	0.778 (0.133)
Mexican American	0.415 (0.107)**	0.527 (0.100)**	0.541 (0.155)*	0.724 (0.167)
Other race/ethnic groups	1.247 (0.367)	0.728 (0.218)	1.286 (0.399)	0.815 (0.265)
Family income quartile				
2			0.783 (0.344)	1.063 (0.230)
3			0.646 (0.169)	0.839 (0.248)
4			0.956 (0.331)	1.220 (0.265)
Head of household education				
Completed high school			1.669 (0.689)	1.775 (0.438)*
> High school			2.109 (0.990)	2.312 (0.436)***

Data are ORs (SEs) for allergy or hay fever diagnosis among unit changes (or categories) of the independent variable. Models are logistic regressions with dependent variable ever diagnosed with allergies or hay fever. Model is estimated among respondents with detected levels of BPA. Model 1 includes age, gender, creatinine, and race as covariates; model 2 includes all of these plus BMI, family income, and educational attainment.

* *p* < 0.05.

** *p* < 0.01.

*** *p* < 0.001.
